# Supplementary material for: Differences in housing wealth between U.S. military service personnel and the Civilian population—Exploring the role of financial stress
Source: PLoS One. 2025 Sep 24;20(9):e0331374. doi: 10.1371/journal.pone.0331374 (PMC12459804; doi:10.1371/journal.pone.0331374)
Supplement: S1 Text — (DOCX) [file pone.0331374.s001.docx]

**S1 Text. Questionnaire text.**

The questions below do not include all questions used in the construction of variables. The full survey questionnaire of the 2022 wave of the Survey of Consumer Finances can be found at: [www.federalreserve.gov/econres/scfindex.htm](http://www.federalreserve.gov/econres/scfindex.htm).

**Focal predictor measures**

(X5906 & X6106) (Have you/Has he/Has she/Has he or she) ever been in the military service?

INCLUDE ONLY SERVICE IN U.S. MILITARY OR NATIONAL GUARD.

1. *YES

5. *NO

0. Inap. (no spouse/partner)

**Stress Variables**

(X7510) Over the past year, would you say that your (family's) spending exceeded your (family's) income, that it was about the same as your income, or that you spent less than your income?

(Spending should not include any investments you have made.) IF DEBTS ARE BEING REPAID ON NET, TREAT THIS AS SPENDING LESS THAN INCOME.

1. *SPENDING SAME AS INCOME

2. *SPENDING SAME AS INCOME

3. *SPENDING WAS LESS THAN INCOME

(X7063) During the past year, have you (or anyone in your family living here) taken out a "payday loan," that is, borrowed money that was supposed to be repaid in full out of your next paycheck?

IF YES: Please do not include personal loans from family members or friends.

1. *YES

5. *NO

(X3004) Now thinking of all the various loan or mortgage payments you made during the last year, were all the payments made the way they were scheduled, or were payments on any of the loans sometimes made later or missed?

1. *All paid as scheduled or AHEAD OF SCHEDULE

5. *Sometimes got behind or missed payments

0. Inap.

(X432) Thinking only about Visa, MasterCard, Discover, American Express cards you can pay off over time, and store cards, do you almost always, sometimes, or hardly ever pay off the total balance owed on the account each month?

1. *Always or almost always

3. *Sometimes

5. *Hardly ever

0. Inap.

(X7775) If tomorrow you experienced a financial emergency that left you unable to pay all of your bills, how would you deal with it? Would you borrow money, would you spend out of savings or investments, would you postpone paying bills, work more or get an extra job, or would you do something else?

1. *BORROW MONEY

2. *SPEND OUT OF SAVINGS/INVESTMENTS

3. *POSTPONE PAYMENTS

4. *CUT BACK

5. *WORK MORE/GET EXTRA JOB

-7. *OTHER

0. Inap.

(X7557) Some people are fully prepared to take financial risks when they save or make investments, while others try to avoid taking financial risks.

On a scale from zero to ten, where zero is not at all willing to take risks and ten is very willing to take risks, what number would you (and your [husband/wife/ partner]) be on the scale?

-1. *NOT AT ALL WILLING TO TAKE FINANCIAL RISKS

1. to 9.

10. *VERY WILLING TO TAKE RISKS

(X7556) Some people are very knowledgeable about personal finances, while others are less knowledgeable about personal finances.

On a scale from zero to ten, where zero is not at all knowledgeable about personal finance and ten is very knowledgeable about personal finance, what number would you (and your [husband/wife/partner]) be on the scale?

-1. *NOT AT ALL KNOWLEDGEABLE ABOUT PERSONAL FINANCE

1. to 9.

10. *VERY KNOWLEDGEABLE ABOUT PERSONAL FINANCE

(X7489) I'd like to start this interview by asking you about your expectations for the future. Over the next year, do you expect the economy to perform better, worse, or about the same as now?

1. *Better

2. *Worse

3. *About the same
