# Supplementary material for: Differences in housing wealth between U.S. military service personnel and the Civilian population—Exploring the role of financial stress
Source: PLoS One. 2025 Sep 24;20(9):e0331374. doi: 10.1371/journal.pone.0331374 (PMC12459804; doi:10.1371/journal.pone.0331374)
Supplement: S1 Table — (DOCX) [file pone.0331374.s002.docx]

S1 Table. Literature review of financial variables.

| Relationship to higher financial stress | Study |
| --- | --- |
| Higher debt or higher debt-to-income ratio | Drentea [1], Bell, Nelson, Spann, Molloy, Britt and Goff [2], Archuleta, Dale and Spann [3], Grable and Joo [4], Xiao, Sorhaindo and Garman [5], O’Neill, Xiao, Sorhaindo and Garman [6] |
| Greater difficulty paying bills on time or collections | Drentea [1], Elbogen, Lanier, Wagner and Tsai [7], FINRA Foundation [8] |
| Carrying a credit card balance | Drentea [1] |
| Lower emergency savings or poor savings behavior | Bell, Nelson, Spann, Molloy, Britt and Goff [2], Hakkio and Keeton [9] |
| Lower willingness to hold risky assets | Hakkio and Keeton [9] |
| Lower subjective (or self-assessed) financial knowledge | Bell, Nelson, Spann, Molloy, Britt and Goff [2] |

Note: A two-step process was used to create a multifaceted financial stress variable. First, using the National Financial Capability Study, a measure of financial anxiety is regressed on a set of seven measures that present dimensions of financial stress and control for key demographic characteristics. In the National Financial Capability Study, anxiety is measured with the question, “How strongly do you agree or disagree with the following statements? Thinking about my personal finances can make me feel anxious.” Responses are coded from 1- Strongly Disagree to 7 – Strongly Agree. Based on a literature review, we selected seven predictor variables (see Figure). Variables were coded in National Financial Capability Study to relate to corresponding variables within the Survey of Consumer Finances.
