# Supplementary material for: Differences in housing wealth between U.S. military service personnel and the Civilian population—Exploring the role of financial stress
Source: PLoS One. 2025 Sep 24;20(9):e0331374. doi: 10.1371/journal.pone.0331374 (PMC12459804; doi:10.1371/journal.pone.0331374)
Supplement: S1 List — (DOCX) [file pone.0331374.s003.docx]

**S1 List. References for literature review.**

1. Drentea P. Age, debt, and anxiety. Journal of Health and Social Behavior. 2000;41(4):437-50. doi: 10.2307/2676296.

2. Bell MM, Nelson JS, Spann SM, Molloy CJ, Britt SL, Goff BN. The impact of financial resources on soldiers’ well-being. Journal of Financial Counseling and Planning. 2014;25(1):41-52.

3. Archuleta KL, Dale A, Spann SM. College students and financial distress: Exploring debt, financial satisfaction, and financial anxiety. Journal of Financial Counseling and Planning. 2013;24(2):50-62.

4. Grable JE, Joo SH. Student racial differences in credit card debt and financial behaviors and stress. College Student Journal. 2006;40(2):400-8.

5. Xiao JJ, Sorhaindo B, Garman ET. Financial behaviours of consumers in credit counselling. International Journal of Consumer Studies. 2005;30(2):108-21. doi: 10.1111/j.1470-6431.2005.00455.x.

6. O’Neill B, Xiao JJ, Sorhaindo B, Garman ET. Financially distressed consumers: Their financial practices, financial well-being, and health. Financial Counseling and Planning. 2005;16(1):73-87.

7. Elbogen EB, Lanier M, Wagner HR, Tsai J. Financial strain, mental illness, and homelessness: Results from a national longitudinal study Medical Care. 2021;59(April (Supplement 2)):S132-S8. doi: 10.1097/MLR.0000000000001453

8. FINRA Foundation. Financial Capability in the United States: 2012 Report of Military Findings. Washington: FINRA Investor Education Foundation; 2013.

9. Hakkio CS, Keeton WR. Financial Stress: What is it, how can it be measured, and why does it matter. Economic Review, Federal Reserve Bank of Kansas City. 2009;94(2):5-50.
