# Supplementary material for: Differences in housing wealth between U.S. military service personnel and the Civilian population—Exploring the role of financial stress
Source: PLoS One. 2025 Sep 24;20(9):e0331374. doi: 10.1371/journal.pone.0331374 (PMC12459804; doi:10.1371/journal.pone.0331374)
Supplement: S2 Table — (DOCX) [file pone.0331374.s004.docx]

**S2 Table. Regression of anxiety on financial stress.**

|  | Coefficient (SE) |
| --- | --- |
| Stress measures: |  |
| Spending to income | 0.43^***^ (0.02) |
| Payday loan | 0.43^***^ (0.04) |
| Late or missed payments | 0.50^***^ (0.03) |
| Carry credit card balance | 0.53^***^ (0.03) |
| Emergency fund | -0.61^***^ (0.03) |
| Willingness to take financial risks | 0.01 (0.01) |
| Self-reported financial knowledge | 0.17^***^ (0.01) |
| NFCS control measures: |  |
| Age | -0.02^***^ (0.00) |
| Male | -0.23^***^ (0.02) |
| Racial/ethnic categories (ref. White) |  |
| Black | -0.33^***^ (0.05) |
| Hispanic | -0.03 (0.05) |
| Asian or other | -0.03 (0.05) |
| Educational categories (ref. Bachelor’s degree or higher) |  |
| Less than high school/GED | -0.23 (0.13) |
| High school/GED | -0.05 (0.03) |
| Some college or associates degree | -0.01 (0.03) |
| Intercept | 4.50^***^ (0.09) |
| Anxiety (mean value (SD), range 1-7) | 4.63 (1.93) |
| N | 18,935 |
| F-test | 577.91^***^ |
| R-squared | 0.30 |

^*^ *p* < 0.05, ^**^ *p* < 0.01, ^***^ *p* < 0.001

Note: In order to link the results of the regression to the main data analysis using the Survey of Consumer Finances, the regression coefficients of the seven predictors, see below table, were multiplied with the corresponding variable in the Survey of Consumer Finances to create the stress composite variable (coefficient ranges: 0.94 to 7.28).

Source: 2021 National Financial Capability Study
