# Supplementary material for: Differences in housing wealth between U.S. military service personnel and the Civilian population—Exploring the role of financial stress
Source: PLoS One. 2025 Sep 24;20(9):e0331374. doi: 10.1371/journal.pone.0331374 (PMC12459804; doi:10.1371/journal.pone.0331374)
Supplement: S3 Table — (DOCX) [file pone.0331374.s005.docx]

**S3 Table. Sample characteristics by military status.**

|  | (1) | | (2) | | (3) | |
| --- | --- | --- | --- | --- | --- | --- |
|  | Korea/Vietnam Era Households | | Post-Vietnam Era Households | | Civilian Households | |
| Variables | Mean | (SD) | Mean | (SD) | Mean | (SD) |
| Homeowner (0/1) | 0.89^***^ | (0.31) | 0.70^**^ | (0.46) | 0.66 | (0.47) |
| Korea & Vietnam Era MSP household (0/1) | 1.00 | (0.00) | - | - | - | - |
| Post-Korea Era MSP household (0/1) | - | - | 1.00 | (0.00) | - | - |
| Stress Variables: |  |  |  |  |  |  |
| Financial stress composite (0-9) | 3.68^***^ | (0.51) | 4.34 | (0.70) | 4.30 | (0.72) |
| Economic expectations, 1-year |  |  |  |  |  |  |
| Worse (0/1) | 0.49 | (0.50) | 0.52^**^ | (0.50) | 0.48 | (0.50) |
| About the same (0/1) | 0.37 | (0.48) | 0.37 | (0.48) | 0.37 | (0.48) |
| Better (0/1) | 0.14 | (0.35) | 0.12^***^ | (0.32) | 0.16 | (0.36) |
| Demographic & socio-economic controls: |  |  |  |  |  |  |
| Age (18-95) | 76.65^***^ | (6.70) | 48.57^***^ | (11.85) | 53.17 | (15.69) |
| Male (0/1) | 0.99^***^ | (0.11) | 0.92^***^ | (0.28) | 0.73 | (0.44) |
| Race |  |  |  |  |  |  |
| White (0/1) | 0.88^***^ | (0.41) | 0.73^***^ | (0.82) | 0.66 | (0.67) |
| Black (0/1) | 0.08^***^ | (0.38) | 0.30^***^ | (0.83) | 0.22 | (0.63) |
| Hispanic (0/1) | 0.05^***^ | (0.34) | 0.25^***^ | (0.82) | 0.20 | (0.62) |
| Asian & other (0/1) | 0.04^***^ | (0.33) | 0.17^*^ | (0.79) | 0.14 | (0.58) |
| Education |  |  |  |  |  |  |
| Did not complete high school/GED (0/1) | 0.05^***^ | (0.22) | 0.02^***^ | (0.15) | 0.10 | (0.30) |
| High school graduate (0/1) | 0.17^**^ | (0.38) | 0.22^*^ | (0.42) | 0.20 | (0.40) |
| Some college or associates degree (0/1) | 0.21 | (0.40) | 0.33^***^ | (0.47) | 0.21 | (0.41) |
| Bachelor's degree or higher (0/1) | 0.57^***^ | (0.50) | 0.42^***^ | (0.49) | 0.49 | (0.50) |
| Married or living with partner (0/1) | 0.76^***^ | (0.43) | 0.74^***^ | (0.44) | 0.61 | (0.49) |
| Children |  |  |  |  |  |  |
| None (0/1) | 0.92^***^ | (0.28) | 0.54^***^ | (0.50) | 0.58 | (0.49) |
| 1-2 (0/1) | 0.08^***^ | (0.27) | 0.38^***^ | (0.49) | 0.33 | (0.47) |
| 3-4 (0/1) | 0.00^***^ | (0.06) | 0.07 | (0.25) | 0.08 | (0.27) |
| 5+ (0/1) | 0.00^***^ | (0.00) | 0.01 | (0.10) | 0.01 | (0.08) |
| Work status |  |  |  |  |  |  |
| Work for other (0/1) | 0.09^***^ | (0.29) | 0.69^***^ | (0.46) | 0.52 | (0.50) |
| Self-employed or partnership (0/1) | 0.24 | (0.42) | 0.14^***^ | (0.35) | 0.22 | (0.41) |
| Retired, disabled, student, homemaker (0/1) | 0.67^***^ | (0.47) | 0.13^***^ | (0.34) | 0.23 | (0.42) |
| Other not working, age<=64 (0/1) | 0.00^***^ | (0.00) | 0.04 | (0.19) | 0.04 | (0.20) |
| Natural log of income (6.07-19.94) | 12.20^***^ | (1.90) | 11.69 | (1.32) | 11.75 | (1.70) |
| N | 301 (6.64%) | | 295 (6.51%) | | 3,936 (86.85%) | |

^*^ *p* < 0.05, ^**^ *p* < 0.01, ^***^ *p* < 0.001

Means comparison tests use Column (3) as a reference category.
