# Supplementary material for: Differences in housing wealth between U.S. military service personnel and the Civilian population—Exploring the role of financial stress
Source: PLoS One. 2025 Sep 24;20(9):e0331374. doi: 10.1371/journal.pone.0331374 (PMC12459804; doi:10.1371/journal.pone.0331374)
Supplement: Attachment — Submitted filename: Manuscript Review 3_16.docx [file pone.0331374.s006.docx]

This manuscript is methodologically sound, concise, and focused. It examines differences in home ownership and housing equity between veterans and civilians and draws from economic frameworks to present clear hypotheses with logical relationships between outcome and explanatory variables. The manuscript, however, lacks context specifying the nature of the problem the authors hope to contribute to and the importance of their findings. Given the methodological strength, I suggest a re-submission.

1. The study analyzes secondary data from multiple sources. Its dependent variables come from the Survey of Consumer Finances, while its explanatory measure of financial stress comes from the National Financial Capability Study. This choice removes common method bias and increases the study’s internal validity. Its other explanatory variable, veteran status, is an objective measure and is unlikely to introduce bias to the results. The authors’ justification for their models and their explanation of their baseline specification is clear and well-reasoned.
2. The authors find support for just one of their four hypotheses – post-Vietnam era military households were associated with lower levels of home equity than civilian households, which the authors argue likely relates to the frequency of geographical re-locations for military members. Also among the key findings is a consistent, perhaps peculiar relationship between economic expectations and homeownership.
   a. Logistic regression models 2 and 3 show that believing the economy would become worse in the next year was associated with a significantly higher likelihood of owning a home, and believing the economy would improve was associated with a significantly lower likelihood of owning a home. This effect partly holds in OLS Model 2, as worse economic expectations were associated with greater home equity.
   b. This finding is intriguing, but the manuscript’s discussion does not interpret its meaning or value. The overall interpretation of the findings is lacking and limits the manuscripts’ contribution. This omission leads me to suggest a second submission. The paper is methodologically sound, and I believe the authors can bolster their discussion section in a revision.
3. While the authors clearly state the value of their findings in contrasting theoretical expectations on the relationship between military status and home ownership, they would benefit from interpreting the results that did yield statistical significance. For example, it is unclear why, in the authors’ minds, lower economic expectations are associated with better housing outcomes or why demographic factors (e.g., race) were consistently associated with negative outcomes. The latter finding may be more obvious, but the manuscript would benefit from a more robust discussion. The study’s rigor is a notable strength, and its methods are presented well, but the lack of interpretation does not allow for a clear conceptual or empirical takeaway.

These major concerns are located in the paper’s back end. The following minor concerns may also merit consideration.

1. The research question on page 4 is presented (grammatically) as a statement. Additionally and more importantly, the statement of the problem on page 4 (“Due to the critical role of housing wealth”) lacks impact. A clearer introduction of the relevant problem and a clearer statement of the first research question would help set a stronger foundation for the methods and results.
2. Perhaps this is a reader issue more than a writer issue, but I did not quite understand section 1.3 of the introduction. The bulk of the preceding content presents evidence about negative financial outcomes for veterans, but section 1.3 ends by vaguely noting financial outcomes have improved. This improvement would seem to contradict Hypothesis 2.
3. The paper is well-written throughout, but like any manuscript, there are minor issues to clean up. I only note the instances below because they did distract from my first read of the manuscript.
   a. The paragraph at the top of page 8 (continued from page 7) is missing a verb after “descriptive analyses.”
   b. At least one in-text citation is presented in parenthesis rather than brackets. I understand this note is pedantic. I include it because it did briefly disrupt the way I read the manuscript. On first glance, the (47) appeared as some sort of specification or clarification rather than a citation.
   c. The “Predictor – financial stress” paragraph in section 2.2 includes an unnecessary indent. Table 1, just below this section, is formatted noticeably differently than the other tables in the manuscript.
